# Optimization of the Hemolysis Assay for the Assessment of Cytotoxicity

**DOI:** 10.3390/ijms24032914

**Published:** 2023-02-02

**Authors:** Ingvill Pedersen Sæbø, Magnar Bjørås, Henrik Franzyk, Emily Helgesen, James Alexander Booth

**Affiliations:** 1 Department of Microbiology, Oslo University Hospital, University of Oslo, Rikshospitalet, 0373 Oslo, Norway; 2 Department of Cancer Research and Molecular Medicine, Norwegian University of Science and Technology, 7491 Trondheim, Norway; 3 Department of Drug Design and Pharmacology, Faculty of Health and Medical Sciences, University of Copenhagen, Jagtvej 162, 2100 Copenhagen, Denmark

**Keywords:** hemolysis assay, hemolysis ratio, cytotoxicity, antimicrobial peptides (AMPs)

## Abstract

In vitro determination of hemolytic properties is a common and important method for preliminary evaluation of cytotoxicity of chemicals, drugs, or any blood-contacting medical device or material. The method itself is relatively straightforward, however, protocols used in the literature vary substantially. This leads to significant difficulties both in interpreting and in comparing the obtained values. Here, we examine how the different variables used under different experimental setups may affect the outcome of this assay. We find that certain key parameters affect the hemolysis measurements in a critical manner. The hemolytic effect of compounds tested here varied up to fourfold depending on the species of the blood source. The use of different types of detergents used for generating positive control samples (i.e., 100% hemolysis) produced up to 2.7-fold differences in the calculated hemolysis ratios. Furthermore, we find an expected, but substantial, increase in the number of hemolyzed erythrocytes with increasing erythrocyte concentration and with prolonged incubation time, which in turn affects the calculated hemolysis ratios. Based on our findings we propose an optimized protocol in an attempt to standardize future hemolysis studies.

## 1. Introduction

Cytotoxicity studies are important during characterization of novel compounds or materials intended for interactions within human biological systems in vivo. Evaluation of the extent to which the compound induces disruption of the membrane of erythrocytes (red blood cells), thereby causing release of cellular content, often constitutes an initial step in such cytotoxicity assessments. The assay used in this context is generally referred to as the hemolysis assay, which has the advantages of being cheap, accessible, and simple to perform. Blood is drawn from a human or an animal, and next, the washed erythrocytes are incubated together with the compound or material under investigation. If the compound causes hemolysis, hemoglobin (along with other cellular constituents) will be released into the supernatant. As hemoglobin has a distinct absorbance spectrum, the degree of hemolysis can be measured in solution by using a standard spectrophotometer or plate reader to provide optical density (OD) values. Finally, the values obtained from samples treated with test compound (OD*_test_*) are normalized relative to positive (100% lysis; OD*_pos_*) and negative (untreated; OD*_neg_*) control samples to give the hemolysis ratio (HR) by using the following equation:

(1) $$HR\left(\%\right)=\frac{{\mathrm{OD}}_{test}-{\mathrm{OD}}_{neg}}{{\mathrm{OD}}_{pos}-{\mathrm{OD}}_{neg}}\times 100\%$$

Spectrophotometric measurement of free hemoglobin is also used in hospital laboratories for evaluation of hemolysis in samples from patients [1,2,3]. Here, hemolysis may occur either in vivo (i.e., within the body) as an indication of a variety of diseases, or in vitro (i.e., outside the body) as a result of improper blood collection or subsequent inadequate sample handling. It is important to identify the cause of hemolysis, since erythrocytes contain constituents, which upon release in vitro, may give rise to false readings of disease indicators present in vivo [4,5,6]. Examples of such constituents are potassium ions, lactate dehydrogenase, and aspartate aminotransferase [6], which are common indicators of diseases such as hyperkalemia, liver damage, or kidney diseases. Thus, assessment of hemolysis is an important tool within many fields, including medical diagnostics [1,2,3], drug development [7,8,9], healthcare technology development [10,11], and life science research [12,13].

Although the hemolysis assay in general appears straightforward, many somewhat deviating protocols are currently used, as is clearly apparent from surveying the literature. These differ by a number of factors, one of the most essential being the source of the blood. Many species have been reported for blood collection, and include human, rabbit, sheep, rat, dog, and horse [14,15,16,17,18,19]. Moreover, the concentration of erythrocytes used for incubation with a test compound varies within the range of 1–4% [15,17,18,20]. There are also examples of labs using whole blood instead of washed erythrocytes [7,21,22,23]. Other variable factors comprise the incubation time (i.e., treatment time with test compound), with examples found between 30 min and 24 h [18,19,24,25] and the wavelength used to measure free hemoglobin (400–600 nm) [15,16,17,18,23,24,25,26,27,28], as well as the type of detergent and concentration used for positive controls. In the latter case, detergents such as Triton X-100, Sodium Dodecyl Sulfate (SDS), and Tween-20 are frequently used to generate 100% lysis at concentrations ranging from 0.1% to 4% [8,16,24,26,27,28,29], or this may be achieved by use of a hypotonic solution such as distilled water to induce osmotic shock that results in hemolysis [13,14,19]. 

It is reasonable to believe that the variations in protocols employed for the hemolysis assay will significantly affect the calculated hemolysis properties (e.g., as HRs), making interpretation of the results difficult. Therefore, we set out to examine the different variables in protocols commonly employed. We find that the species that the blood originated from, detergents used for positive controls, erythrocyte concentration, and incubation time all have significant effects on the measured degree of hemolysis. We conclude by suggesting an optimized protocol enabling standardized hemolysis measurements that facilitate comparative analysis of data.

## 2. Results

### 2.1. Species-Dependent Effects on Hemolysis

**Figure 1:**
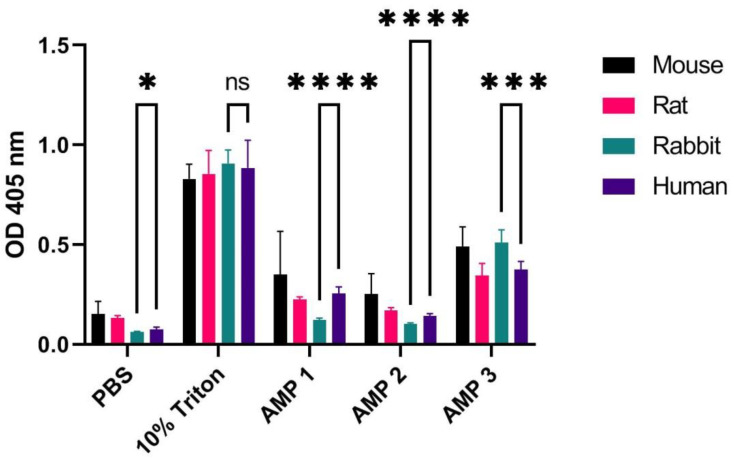
OD measurements at 405 nm (Y-axis) of free hemoglobin in a 1% erythrocyte solution originating from a mouse, rat, rabbit, and human, incubated for 60 min at 37 °C with PBS (negative control), 10% Triton X-100 (positive control), or AMPs 1, 2 or 3 (at concentrations of 100 µM). Average values from three experimental replicates, each containing two technical replicates, are presented with error bars (SD) included in plots. Significantly different data as defined from unpaired *t*-test is indicated by asterisks for comparison of human and rabbit samples (*p*-values: * < 0.05 *** < 0.001 **** < 0.0001 ns: non-significant). See Table S1 for *p*-values from comparison of all species.

One of the most obvious factors that may affect the outcome of hemolysis assays is the source of the blood. In the literature, we find reports on hemolysis with a large variability with respect to the species origin of the blood, ranging from human to horse [14,15,17,18,19]. In the present work, we examined whether the hemolytic effect of three in-house antimicrobial peptides (AMPs) differ in blood originating from human, rat, rabbit, and mouse. The AMPs, hereafter denoted as AMPs 1, 2, and 3, have in vitro bactericidal activity against a range of Gram-positive and Gram-negative pathogens (unpublished results), and were chosen because of observed low (AMP 2), medium (AMP 1), and high (AMP 3) hemolytic properties. AMPs 1, 2, and 3 have lengths of 29, 20, and 26 amino acids, and hydrophobicity of 48%, 40%, and 46% (as calculated by using peptide2.com), respectively. For the hemolysis assay, we used 1% washed erythrocytes from the above-mentioned species, incubated for 60 min at 37 °C with peptide solutions and positive (10% Triton X) and negative (phosphate-buffered saline, PBS, pH 7) controls, after which the optical density (OD) was measured at 405 nm to detect the amount of hemoglobin released (see Materials and Methods and figure legend for details). These parameters were chosen because of findings presented and discussed later in this study. We find that all three AMPs exert species-dependent effects on erythrocyte lysis, as observed from OD measurements (Figure 1) and calculated hemolysis ratios (HRs) (Table 1). The most significant differences in OD measurements of hemoglobin were found when comparing human and rabbit erythrocytes (*p*-values of unpaired *t*-tests are indicated in Figure 1; see Table S1 for *p*-value comparisons for all species). For HR values, the largest discrepancy was found for AMP 1, which produced over four times higher HR in the mouse sample as compared to that of the rabbit sample (Table 1). This indicates that the choice of species used for hemolysis assays may affect the results to a surprisingly large extent. We also noticed that blood drawn from animals of smaller size (rat and mouse) had a higher degree of hemolysis in the negative controls (1% erythrocytes incubated with PBS), indicating that in these cases, there was a significant degree of pre-analyte hemolysis.

### 2.2. Blood Drawn from Different Human Individuals Appears to Respond to Hemolytic Agents to a Similar Extent

**Figure 2:**
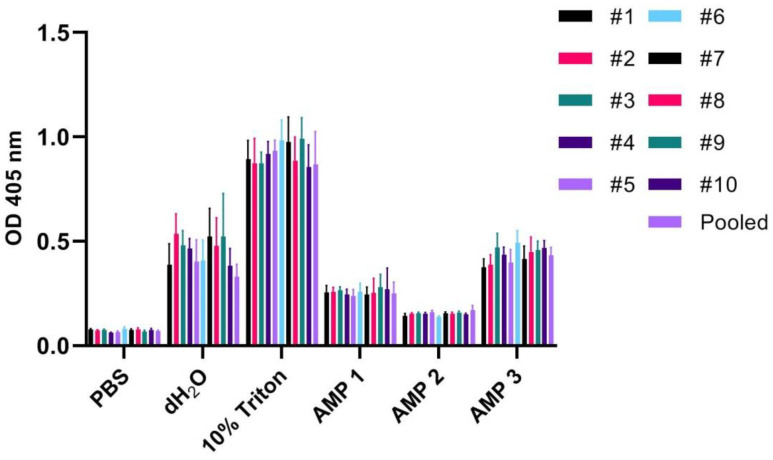
OD measurements at 405 nm (Y-axis) of free hemoglobin in 1% erythrocyte solutions originating from 10 different human individuals, as well as blood pooled from all individuals, incubated for 60 min at 37 °C with PBS (negative control), 10% Triton X-100 (positive control), dH_2_O, or AMPs 1, 2, or 3 (100 µM). Average values from three experimental replicates, each containing two technical replicates, are presented with error bars (SD) included in plots.

Since we found that the species origin of blood used for hemolysis assays affects the hemolytic response to such a significant degree, we speculated whether this might also be true for blood originating from different human individuals. Hence, blood samples were drawn from 10 healthy human volunteers, and then the assay was repeated as described for Figure 1. Here, we also included distilled water (dH_2_O), which causes hemolysis through osmotic shock, and often is used as the positive control in hemolysis assays [13,14,19]. For these 10 individuals, we found that the hemolytic responses appeared similar towards each of the peptides, PBS, and hemolyzing agents (one-way ANOVA test: *p*-value > 0.9999) (Figure 2). Slight (but non-significant) differences were found for dH_2_O and the positive control Triton X-100, of which the latter resulted in some variations also when calculating the HR values (Table 2). We also checked the erythrocyte counts in the samples prior to assay start, and these were found to be reasonably similar between individuals and experimentation day, with variations within 3 to 6 million cells in a 1 mL 1% suspension (Figure S1). Finally, we examined whether the type of anticoagulant used in blood collection tubes (heparin vs. sodium citrate) had an influence on the results, but it was found not to be the case (Figure S2).

### 2.3. The Erythrocyte Concentration in Samples Affects Measurements of Free Hemoglobin

**Figure 3:**
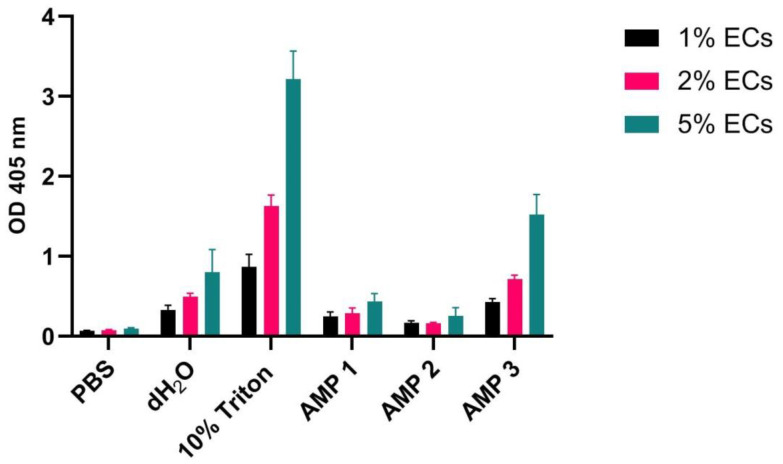
OD measurements at 405 nm (Y-axis) of free hemoglobin in 1%, 2%, and 5% erythrocyte (EC) solution of pooled blood originating from 10 different human individuals, incubated for 60 min at 37 °C with PBS (negative control), 10% Triton X-100 (positive control), dH_2_O, or AMPs 1, 2, or 3 (100 µM). Average values from three experimental replicates, each containing two technical replicates, are presented with error bars (SD) and included in plots.

As mentioned in the introduction, variations in the concentration of washed erythrocytes, used for hemolysis testing, are commonly reported in the literature [15,17,18,20]. Therefore, we next investigated the effect of erythrocyte concentration on hemolysis measurements. Expectedly, it was found that OD measurements of free hemoglobin increases with increasing concentration of erythrocytes in all samples, except for the negative controls (Figure 3). The ratio between Triton X-100 and AMP 3 measurements remained constant with increasing erythrocyte concentration (~0.5), and correlations were close to linear (Goodness of fit test R^2^ 94.7% and 91.3%, respectively), resulting in only minor effects on the calculated HR values (Table 3). However, for AMPs 1 and 2, as well as distilled water, the increase in erythrocyte concentration resulted in decreased HR values (Table 3).

### 2.4. Sample Incubation Time Affects the Degree of Hemolysis

**Figure 4:**
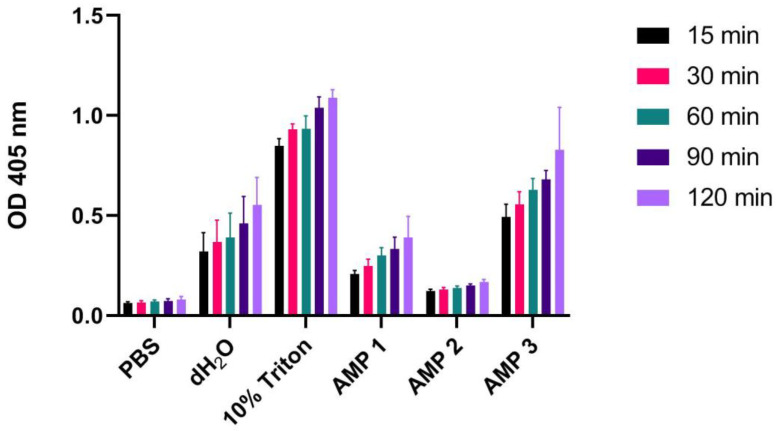
OD measurements at 405 nm (Y-axis) of free hemoglobin in a 1% human erythrocyte solution incubated for 15, 30, 60, 90 or 120 min at 37 °C with PBS (negative control), 10% Triton X-100 (positive control), dH_2_O or AMPs 1, 2 or 3 (100 µM). Average values from three experimental replicates, each containing two technical replicates, are presented with error bars (SD) included in plots.

To our knowledge, there is no consensus on which sample incubation time to use in hemolysis assays. While most labs incubate the erythrocytes with test compound for 60 min, others use shorter [18] or longer [24,25] incubation times. In order to assess the effect this has on the measured hemolysis, we tested incubation times of 15, 30, 60, 90, and 120 min on 1% washed human erythrocytes. From these results, we found a very clear trend in which the degree of released hemoglobin increases linearly with increasing incubation time, except for the negative control (Figure 4). This in turn also affects the calculated HR values (Table 4). There are negligible differences between time points 15 and 30 min, however, increasing incubation up to 120 min produces a 1.43- and 1.65-fold difference for dH_2_O and AMP 1, respectively, relative to the shortest incubation times. AMP 2, on the other hand, was less sensitive to time-dependent effects on hemolysis (Table 4). This arises from the fact that AMP 2/Triton X-100 ratios remained almost constant with increasing incubation time (i.e., a ratio of ~1).

### 2.5. Large Variations in Hemolytic Response to Different Detergents Used as Positive Controls

**Figure 5:**
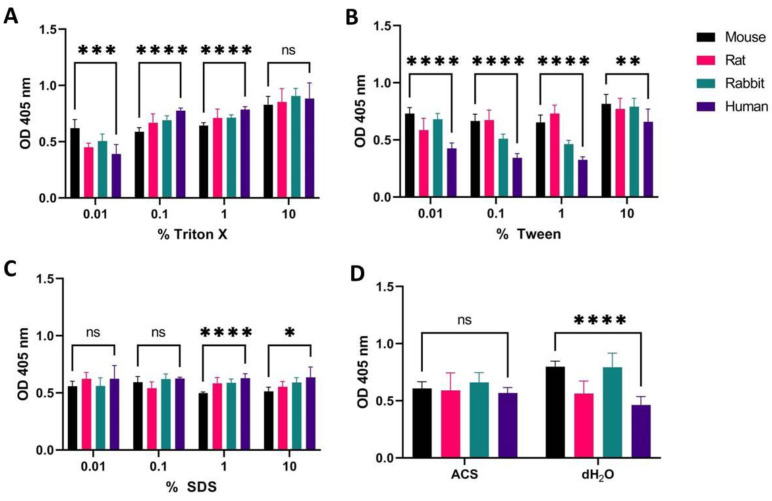
OD measurements at 405 nm (Y-axis) of free hemoglobin in a 1% erythrocyte solution sourced from a mouse, rat, rabbit, and human treated with different concentrations of Triton X-100 (**A**), Tween (**B**), and SDS (**C**), as well as ACS or dH_2_O (**D**). Erythrocytes were incubated for 60 min at 37 °C. Average values from three experimental replicates, each containing two technical replicates, are presented with error bars (SD) included in plots. Significantly different data as defined from unpaired *t*-test is indicated by asterisks (*p*-values: * < 0.05 ** < 0.01 *** < 0.001 **** < 0.0001 ns: non-significant).

In order to calculate the degree of hemolysis for test compounds, positive and negative controls are included to normalize the results by using Equation (1). In this context, it is crucial that the positive control sample in fact contains cells that are hemolyzed as completely as possible, so that the maximum amount of hemolysis is well defined. Since we found in the literature that different types of detergents over a range of concentrations were employed for generation of positive controls, we wished to determine whether these types and amounts of detergents are sufficient to produce full lysis of all erythrocytes present in the sample. Comparative plots of mouse, rat, rabbit, and human erythrocytes, treated with Triton X-100, Tween, or SDS at concentrations from 0.01 to 10% as well as dH_2_O, were constructed (Figure 5). Higher detergent concentrations were avoided because of the viscous nature of pure detergents, making it difficult to pipette accurate amounts into solution. Ammonium chloride solution (ACS) was also included because of its reported use as a specific erythrocyte-hemolyzing agent [30]. From these plots, it is clear that not all detergents are suitable for use as positive controls, and this is again dependent on detergent concentration and the species origin of the blood. The highest OD measurements, representing the highest degree of hemolysis, were around 0.8–0.9 as found for 10% Triton X-100, 10% Tween, and dH_2_O (Figure 5A,B,D). However, dH_2_O only produced a high level of hemolysis for erythrocytes from mouse and rabbit, whereas the values for rat and human were 1.33-fold and 1.6-fold lower, respectively (Figure 5D and see Table 2 for *p*-values). Concentrations below 10% Tween also produced a highly species-dependent variation, in which OD measurements of human erythrocytes were more than two times lower than those of mouse and rat (in 0.1% and 1% Tween, Figure 5B). For SDS the concentration appeared to be less critical, however, measurements never increased above 0.6 for these concentrations, thus not representing full hemolysis (Figure 5C). Plots presenting higher resolution of detergent concentrations (0.001% to 10%) showed that values for SDS in fact peak at 0.02% (OD 0,8), and then decrease at higher concentrations for human erythrocytes (Figure S3).

Under the experimental setup presented above, 10% of Triton X-100 was found to give the overall highest absorbance measurements with the lowest variation between species (see Figure 5 and Table S2 for *p*-values).

### 2.6. Use of Whole Blood vs. Washed Erythrocytes in Hemolysis Assays: Choice of Wavelength and Effect on Hemolysis

In the present work, we have so far discussed results from experiments based on washed erythrocytes. However, some labs use whole blood for hemolysis assays [7,21,22,23]. Whole blood has very different optical properties as compared to washed erythrocytes, and a range of different wavelengths have been employed for measurements of hemoglobin in both whole blood [7,21,23,31] and in suspensions of washed erythrocytes [15,17,25,26,28]. Hemoglobin is reported to have distinct absorbance peaks around 410, 545, and 570 nm, whereas bilirubin, which is present in whole blood, absorbs at 450 nm, but affects OD readings within the entire range of 350–550 nm [32]. Turbidity in the sample also affects measurements at the lower wavelengths to a larger extent than at the higher wavelengths [32]. We therefore examined how the use of different wavelengths affects measurements of both whole blood and washed erythrocytes.

**Figure 6:**
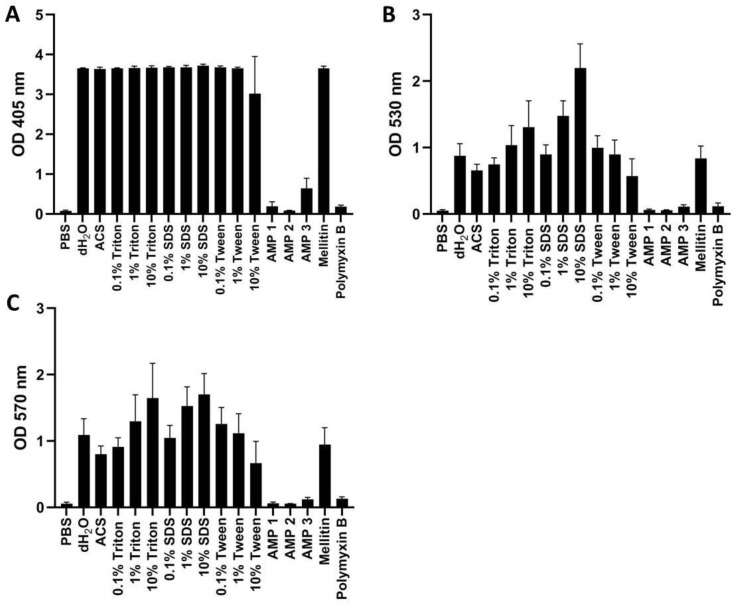
OD measurements of free hemoglobin in human whole blood at 405 nm (**A**), 530 nm (**B**), or 570 nm (**C**). Samples were treated with PBS (negative control), AMPs 1, 2, 3, melittin, or polymyxin B (all at 100 μM), as well as with dH_2_O, ACS, or different concentrations of Triton X-100, Tween, or SDS. All samples were incubated for 60 min at 37 °C. Average values from three experimental replicates, each containing two technical replicates, are presented with error bars (SD) included in plots.

OD measurements of human whole blood treated with hemolyzing agents or AMPs at 405 nm (A), 530 nm (B), and 570 nm (C) were recorded (Figure 6) and HRs calculated (Table 5). For comparison, we also included the two previously well-characterized AMPs melittin and polymyxin B, which exert high and low cytotoxicity, respectively [9,33]. From these results, it can be seen that measurements at 405 nm are inappropriate for whole blood, as the signal becomes saturated for all positive controls, independent of detergent concentration (Figure 6A). From 530 and 570 nm measurements, the expected concentration-dependent increases in absorbance were seen for Triton X-100 and SDS (Figure 6B,C). The opposite was observed for Tween, for which hemolysis in whole blood appears to decrease with increasing Tween concentration. This effect was caused by an observed tendency of the blood to clot at higher Tween concentrations. The clotting effect was also observed for melittin. The use of 10% SDS produced the highest absorbance for measurements at both 530 and 570 nm (2.2 and 1.7, respectively). We repeated the experiment with rabbit whole blood to examine a possible species-dependent difference also in this context (Figure S4 and Table S3). The same trend was seen here, i.e., (i) 405 nm measurements are saturated, and (ii) absorbance decrease with increasing Tween concentration at 530 and 570 nm. However, 10% Triton X-100 appears to result in higher hemolysis than 10% SDS, when measuring at 570 nm in rabbit whole blood (Figure S4C).

**Figure 7:**
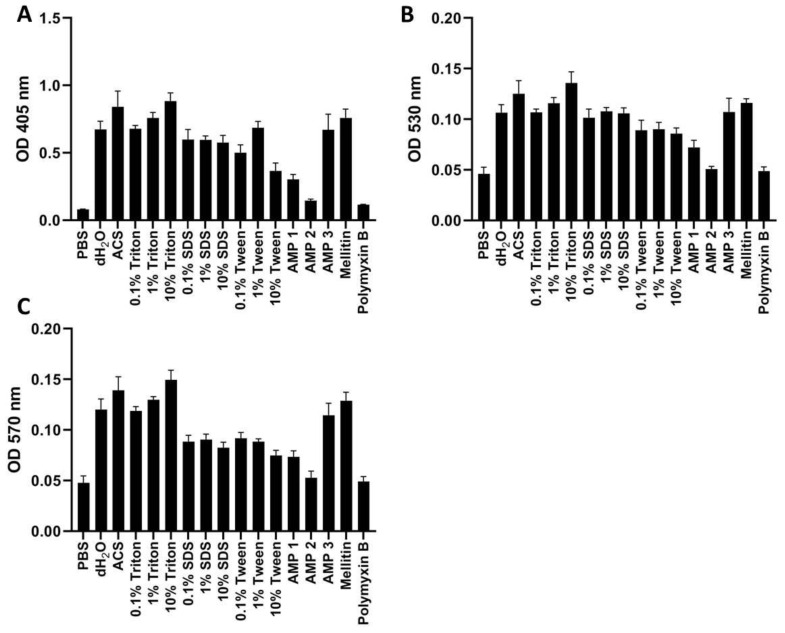
OD measurements of free hemoglobin in 1% washed human erythrocytes at 405 nm (**A**), 530 nm (**B**), or 570 nm (**C**). Samples were treated with PBS (negative control), AMPs 1, 2, 3, melittin or polymyxin B (all at 100 μM), as well as with dH_2_O, ACS, or different concentrations of Triton X-100, Tween, or SDS. All samples were incubated for 60 min at 37 °C. Average values from three experimental replicates, each containing two technical replicates, are presented with error bars (SD) included in plots.

We next investigated how hemoglobin measurements were affected by the use of different wavelengths on washed human erythrocytes (Figure 7). We found that OD measurements at 530 and 570 nm (Figure 7B,C) were very low in comparison to those recorded at 405 nm (Figure 7A), with the highest values produced in the presence of 10% Triton X-100 being 0.14 and 0.15 at 530 and 570 nm, respectively, vs. 0.88 at 405 nm. Values for the negative control were, on the other hand, high relative to the positive control at 530 and 570 nm, producing only a 3-fold difference between measurements of positive and negative control. At 405 nm, the positive control values were 11-fold higher than the negative control, giving a larger window of separation for sample measurements. When normalizing the data to obtain the HR values, there is only a slight decrease in values with increasing wavelength (Table 6). OD measurements at 405, 530, and 570 nm were also obtained for washed erythrocytes from the mouse, rabbit, and rat, showing similar trends as for washed human erythrocytes (Figure S5).

When examining the hemolytic effects for AMPs 1, 2, and 3 more closely, it was striking that they caused very low hemolysis in whole blood as compared to what was found in suspensions of washed erythrocytes (cf. Figure 6 and Figure 7). After calculating the HR values in human whole blood when using 10% SDS as positive control, we found that the HR for AMPs 1, 2, and 3 were reduced to 0.4, 0.2, and 2.7% hemolysis when measured at 530 nm, and to 0.4, −0.1 and 3.8% when measured at 570 nm (Table 5). These results correspond to more than 56-, 41-, and 27-fold reductions in the HR for AMPs 1, 2, and 3, respectively, in comparison with HR values obtained with washed erythrocytes measured at 405 nm (Table 6). Distilled water, ACS, and melittin also showed decreased hemolytic activity in whole blood in comparison to the findings for washed erythrocytes, albeit at a lower ratio (cf. Table 5 and Table 6). For polymyxin B, this effect was not seen, as the HR values were similar in whole blood and washed erythrocytes.

For rabbit whole blood, the reduction in hemolytic effect was less dramatic for the AMPs, with AMP 2 and AMP 3 exhibiting 23- and 4-fold decreased effects, respectively. AMP 1, on the other hand, exhibited slightly increased hemolytic effect in rabbit whole blood (cf. Tables S3 and S4).

### 2.7. Plasticware Used for Incubation 

**Figure 8:**
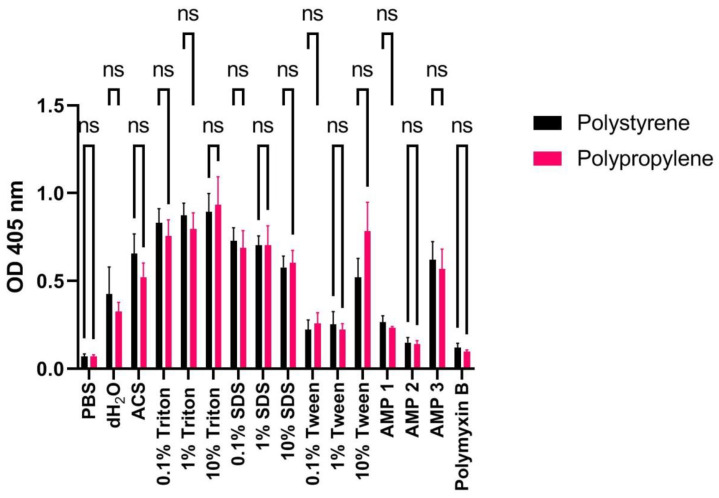
OD measurements at 405 nm (Y-axis) of free hemoglobin in a 1% human erythrocyte solution incubated with PBS, dH_2_O, ACS, Triton X-100, SDS, Tween, AMPs 1, 2, or 3 (100 μM), or polymyxin B (100 μM) for 60 min at 37 °C in polystyrene (black bars) or polypropylene (red bars) tubes. Average values from three experimental replicates, each containing two technical replicates, are presented with error bars (SD) included in plots. Significantly different data as defined from paired *t*-test is indicated by asterisks (ns: non-significant).

Adsorption of detergents or compounds onto plastic material used during incubation may potentially affect the degree of erythrocyte hemolysis. In particular, this effect has been reported for studies involving peptides [34]. The two types of plastic normally used in labs are polystyrene (PS) and polypropylene (PP). Therefore, we compared the hemolytic effect of our AMPs and detergents when incubated with washed human erythrocytes in PS vs. PP tubes for 60 min at 37 °C. We only find minor, non-significant differences (Figure 8; *p*-values from paired *t*-tests shown in the figure). Similarly, differences were not large when calculating the HR values using 10% Triton X-100 as positive control (Table 7). 

### 2.8. Downscaling of Sample Volumes to 96-Well Format Retains Hemolysis Ratio Data Quality

**Figure 9:**
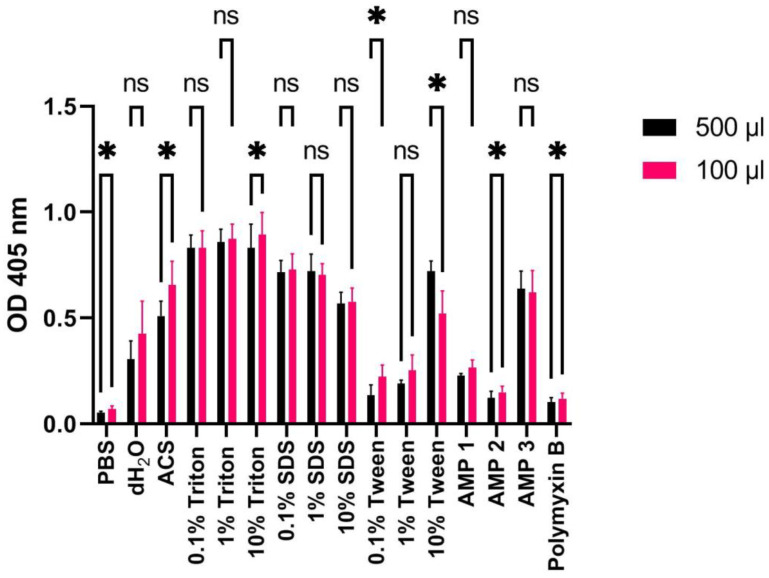
OD measurements at 405 nm (Y-axis) of free hemoglobin in a 1% human erythrocyte solution incubated with PBS, dH_2_O, ACS, Triton X-100, SDS, Tween, AMPs 1, 2, or 3 (100 μM), or polymyxin B (100 μM) for 60 min at 37 °C using high (500 μL: black bars) or low (100 μL; red bars) volumes. Average values from three experimental replicates, each containing two technical replicates, are presented with error bars (SD) included in plots. Significantly different data as defined from paired *t*-test is indicated by asterisks (*p*-values: * < 0.05 ns: non-significant).

For hemolysis assays it would be advantageous to use low volumes in 96-well plates in order to reduce the amount of blood and compound needed, and to more efficiently perform pipetting steps. In the literature, significant volume variations are also reported in hemolysis assays, ranging from 100 μL to 2 mL [8,18,24,35,36,37,38]. Therefore, we examined whether small (100 µL) or higher (500 µL) volumes during the incubation step affect the results significantly. From Figure 9, it can be seen that variations in OD measurements at 405 nm on human washed erythrocytes are relatively small for most hemolyzing agents (*p*-values indicated in figure). The calculated HR values reflect the low variations accordingly (Table 8). For samples marked with significant differences (*p*-values between 0.05–0.01), 100 μL volumes produced higher hemolysis than 500 μL volumes for some of the most relevant samples (10% Triton X-100, AMP 2, and PMB).

## 3. Discussion

In the present work, we show that the large variability found in experimental setup for assessment of hemolysis in the literature may lead to spurious and incomparable results. The most pronounced discrepancies were found when comparing the following variables: (i) species origin of erythrocytes (Figure 1), (ii) erythrocyte concentration (Figure 3), (iii) incubation time (Figure 4), and (iv) type of detergent used as positive control (Figure 5). We also found that testing using whole blood may result in significantly reduced hemolytic activity of test compounds (Figure 6) in comparison to results obtained with washed erythrocytes (Figure 7). The aim of this work is to convey increased awareness concerning these parameters, and to suggest a higher degree of standardization for the hemolysis assay.

### 3.1. Choosing Species Origin of Erythrocytes

The large differences in hemolytic activity of compounds and detergents towards erythrocytes from different species (Figure 1) likely stem from differences in the composition of proteins and lipids in the erythrocyte membranes [39], as discussed in Greco et al. [15]. This includes expression of different sets of transmembrane proteins that pump ions or water, which give them increased or decreased susceptibility towards lysis [40,41,42]. Differences could also arise from variations in hemoglobin content in cells, however, hemoglobin content is proportional to cell size and differences appear to be small among mammalian species [43].

Most compounds that undergo cytotoxicity assessments are ultimately intended for use in humans. However, in order to reach clinical trials, it is required that efficacy and safety is demonstrated in vivo in animal models [44], typically being a mouse, rat, monkey, guinea pig, or pig. Therefore, it is advisable that this is kept in mind already at the stage of cytotoxicity assessment in vitro, and that testing is performed by using both blood from humans and from the species intended for initial studies in vivo. We also noted that blood drawn from smaller animals (mouse and rat) had a higher degree of pre-analyte hemolysis (Figure 1; negative controls). This could be caused by the use of thinner needles during phlebotomy (for mice), which may physically rupture erythrocytes [4], and/or by the blood collection procedure itself (heart phlebotomy).

When testing blood from different human individuals, we found no clearly significant differences in erythrocyte count or hemolytic activity of the tested detergents and compounds (Figure 2). However, the standard deviations were relatively large for the most hemolytic compounds, which may indicate more subtle underlying differences in the dataset. It may be necessary to include an increased number of experimental replicates to make unambiguous conclusions. Thus, the 10 individuals included might not be sufficient to detect outliers. Undiscovered conditions causing anemia may for example generate a lower erythrocyte count in certain individuals [45]. In the present work, we did not compare blood from different human individuals for whole-blood experiments. Here, individual variations in factors present in blood that interact with test compounds may contribute to variations in the hemolytic response of erythrocytes [11,46,47], as also discussed below.

### 3.2. Washed Erythrocytes vs. Whole Blood

Hemolysis assays are performed using both washed erythrocytes and whole blood [7,14,15,18,21,23]. However, the hemolytic response resulting from treatment with test compounds can be dramatically different under these two conditions, as seen for our AMPs, which produced up to 56-fold lower hemolysis ratios in whole blood as compared to those obtained in washed erythrocytes (Table 6 and Table 7). Surprisingly, one peptide (AMP 1) exhibited higher hemolysis in rabbit whole blood (Table S3) as compared to washed rabbit erythrocytes (Table S4). The reason for this could be a lower tendency of AMP 1 to bind to constituents present in rabbit whole blood (for example serum albumin), thereby freeing more peptide molecules for activity against erythrocytes. We also found that certain highly hemolytic agents, such as Tween (at high concentrations) and melittin, have a tendency to cause blood clotting, which makes the measurements inaccurate. 

On one hand, it can be argued that the use of whole blood is more physiologically relevant for drugs intended for intravenous injection or for materials directly contacting blood. On the other hand, the hemolysis assay is generally intended as a rapid test for evaluation of lytic interactions with mammalian membranes. Since blood contains numerous factors that may adsorb or degrade peptides or other drugs or materials [11,46,47], the use of whole blood may therefore disguise the hemolytic properties of the drug hit or candidate. Therefore, we advise that the intended application of the test compound should be taken into consideration when choosing whether to use full blood or washed erythrocytes. In some instances, it may be pertinent to perform both variants of the assay. Under conditions in which assessment of hemolytic effect in whole blood is desirable, we suggest measuring at wavelengths above 550 nm to avoid contributions from bilirubin and turbidity (Figure 6 and [32]). Because of the observed blood clotting, we also suggest avoiding the use of Tween as positive control, and to be aware that certain highly hemolytic compounds may cause similar clotting effects (as found here for melittin). For washed erythrocytes, we advise measurements at 405 nm in order to ensure the highest dynamic range (see Figure 7). It should be noted that different spectrophotometers may exhibit somewhat different sensitivities, and that the cuvette dimensions will also affect the readings according to the Beer-Lambert law [48].

### 3.3. Erythrocyte Concentration and Incubation Time

Erythrocyte concentrations in samples as well as the sample incubation time both affect the measurement of free hemoglobin. The measured values simply increase with increasing erythrocyte concentration or incubation time (Figure 3 and Figure 4, respectively). However, the only cases in which these parameters do not affect the calculated HR values are when hemoglobin measurements increase by the same factor for the positive control and the test compound. This was seen for AMP 3 with varying concentrations of erythrocytes (Table 3), and for AMP 2 with varying incubation times (Table 4). In all other cases, the HR values were affected by alterations in the erythrocyte concentration and incubation time. The erythrocyte concentration effect bears similarities to the so-called inoculum effect for peptides and antibiotics, in which the inhibitory concentration of a peptide or antibiotic increases as the initial experimental inoculum is raised [49]. In our assays, the compounds are likely present at lower concentrations than needed to saturate the cell surfaces, and thus the HR becomes less dependent of the number of available erythrocytes. The incubation time effect could, on the other hand, be a kinetic effect that reflects the time required for the AMPs to interact with the membrane and lyse the erythrocytes. The slower the interaction kinetics, the less dependent the HR will be on incubation time. Since it is very difficult to predict whether test compounds will behave as positive controls, both of these variables should be kept constant in order to facilitate comparison of values between labs. The most common erythrocyte concentration found in the literature is 1%, while the most common incubation time is 60 min. We, therefore, suggest using these parameters for a standardized hemolysis protocol. 

It is also worth mentioning that a similar effect occurs when it comes to the storage time of the washed erythrocytes. Longer storage times prior to experimentation produce a higher degree of erythrocyte fragility and hemolysis (Figure S6), indicating that the blood should optimally be used as soon as possible after its collection.

### 3.4. Choosing the Most Efficient Detergent as a Positive Control

Detergents commonly used to generate positive hemolysis controls (SDS, Tween, and Triton X-100), as well as distilled water, were found to produce varying degrees of hemolysis (Figure 5 and Figure S3). These effects were dependent on detergent concentration and on erythrocyte species. As already discussed above, species-dependent effects are likely caused by differences in membrane composition, conferring different degrees of resilience towards hemolysis. Nonetheless, it is important to recognize these differences, because the use of an improper positive control may result in large discrepancies in the calculated HR values (Equation (1)). For example, distilled water produced a high degree of hemolysis for mouse and rabbit erythrocytes, whereas its effect was considerably less pronounced for human and rat erythrocytes (Figure 5). Differences regarding the concentration of detergent were less surprising, since the measurements generally increased with increasing detergent concentration, as reported previously [50]. However, an interesting observation for SDS was that the OD measurements peaked at 0.02%, and then decreased at higher concentrations (Figure S3). Upon reviewing the literature, we found that SDS interacts with hemoglobin to produce hydrogen peroxide, which results in degradation of hemoglobin [13]. The decrease in hemoglobin measurements at concentrations >0.02% could, therefore, be attributed to this effect. Thus, we suggest avoiding the use of SDS for positive controls in hemolysis assays. In contrast 10% Triton X-100 gave overall high OD measurements of hemoglobin with non-significant differences between species (Figure 5 and Figure S3). Therefore, this detergent is proposed for use in a standardized hemolysis assay protocol.

### 3.5. Downscaling of Experiments to 96-Well Plates

Our results indicate that downscaling of hemolysis experiments to 96-well plates only affects measurements to a minor extent (Figure 9). Certain samples, such as erythrocytes incubated with AMP 2 or PMB, showed slightly increased hemolysis in 100 μL volumes, indicating that care should be taken when comparing data from experiments conducted with higher volumes. The use of lower volumes eases experimentation since both less blood and a potentially expensive test compound are required. Moreover, pipetting steps of a larger number of samples with replicates are readily performed by using multichannel pipettes or robotics. When it comes to the type of plastic to use in 96-well plates for incubation of erythrocytes with detergents and compounds, we do not find any significant differences (Figure 8). From these data we cannot predict how other types of compounds will behave, however, PP is reported to have a lower binding propensity for polar molecules such as DNA, proteins, and peptides [34,51,52]. As discussed in Citterio et al. [52], this effect only becomes critical at low micromolar concentrations, since the fraction of adsorbed compound then is so large that it severely diminishes the amount of free AMP available in solution for membrane interactions. 

### 3.6. Other Remarks

Here, we do not consider whether evaluation of cytotoxicity by using hemolysis assays in fact constitutes a viable way to predict cytotoxicity in vivo, which is a subject debated elsewhere [15,33,53]. However, we carried out an assessment of whether the degree of hemolysis in fact correlates with cell viability by performing flow cytometry of treated erythrocytes stained with an amine-reactive live/dead fluorescent probe (Figure S7). Assuming all intact cells are alive and all dead cells are lysed, and thus not quantified, one would expect low fluorescence for all counted cells. For cells treated with PBS, dH_2_O, or AMP 2, we found that this indeed is the case (Figure S7). AMP 3, however, showed surprisingly high fluorescence intensity measurements per cell (Figure S7A), which indicates that most of the intact cells left in this sample were in fact dead. Such effects will depend on the compound tested, and it may be important to keep in mind when assessing cytotoxicity by using the hemolysis assay.

PROPOSED STANDARDIZED PROTOCOL FOR THE HEMOLYSIS ASSAY

*Material list:*

- Triton X-100 (Sigma-Aldrich, Saint-Louis, MO, USA, T8787)
- Phosphate-buffered Saline (PBS) pH~7
- LH Lithium Heparin tubes (4 mL; Greiner Bio-One, Frickenhausen, Germany, 454029) or Sodium Citrate 3.2% tubes (3 mL; Greiner Bio-One, Frickenhausen, Germany, 454334)
- 96-well Polypropylene PCR plate (VWR, Radnor, PA, USA, 82006-636 or similar). For incubation step.
- 96-well plate with flat, transparent bottom (Anicrin, Moglianese, Italy, A013418 or similar). For OD measurements in the plate reader.

*Procedure:*

1. Collect blood in heparin or sodium citrate tubes and immediately centrifuge at 1700× *g* for 5 min. Avoid using needles above 23 G in order to minimize pre-analyte hemolysis.
2. Remove the supernatant by aspiration and wash the erythrocytes by adding 2 mL of PBS pH~7. Centrifuge at 1700× *g* for 5 min. Repeat the washing step three times or until supernatant is clear.
3. Remove supernatant and dilute the erythrocyte pellet 1:100 in PBS pH~7 to obtain a 1% erythrocyte suspension.
4. Mix 50 µL of the 1% erythrocyte suspension with 50 µL of test compound in a 96-well polypropylene plate with conical wells (PCR plate). The conical shape makes it easier to pipette the supernatant in the next step. Use 10% Triton X-100 as a positive control and PBS pH~7 as a negative control in identical volumes as test compounds. NB: 10% Triton X-100 solution should be made by weighing (*w*/*v*) to obtain an accurate concentration.
5. Incubate the plate at 37 °C for 60 min.
6. Centrifuge the plate at 1700× *g* for 5 min.
7. Transfer 50 µL of the supernatant to a transparent, flat-bottom 96-well plate and measure absorption at 405 nm in a plate reader.

The experiment should contain at least two technical replicates (i.e., two wells per sample) and be repeated at least three times for statistical power.

## 4. Materials and Methods

### 4.1. Blood Collection

A VACUETTE butterfly needle 23 G (blue) 19 cm tube with luer adapter (Greiner Bio-One, Frickenhausen, Germany) was used for phlebotomy of human blood from peripheral veins of the arm. 

Blood from mice, rats, and rabbits was provided by the Department of Comparative Medicine at Oslo University Hospital (Marianne Aannestad). For mice and rats, the blood was collected from the heart by using 25 G and 21 G needles, respectively, whereas for rabbits, the blood was collected by free bleed through the ear using a yellow neoflon (24 G). All animals were wild type and anesthetized prior to the procedure.

All blood was collected in Lithium Heparin tubes 4 mL (Greiner Bio-One, Kremsmünster, Austria) or Coagulation sodium citrate 3.2% tubes 3 mL (Greiner Bio-One). 

### 4.2. Antimicrobial Peptides

Antimicrobial peptides 1, 2, and 3 were designed based on peptide sequences from toxin-antitoxin systems in Gram-negative bacteria and optimized to improve solubility by substitution of highly hydrophobic residues (Table S5). AMP 2 contained an N-terminal hexanoic acid moiety, whereas all peptides contained amidated C-termini. These three peptides were synthesized by Genscript Inc. (Piscataway, NJ, USA), and stock solutions of 1 mg/mL were made from the purchased powders. Polymyxin B was purchased from Sigma Aldrich (P4932) and dissolved in dH_2_O to obtain a 50 mg/mL stock solution. Melittin was synthesized at the University of Copenhagen and dissolved in PBS to obtain a 3 mg/mL stock solution. All peptides were diluted to yield a 100 μM final concentration in the hemolysis assays.

### 4.3. Preparation of Detergent Solutions for Positive Controls

Triton X-100 (T8787) and Tween-20 (P2287) detergents were purchased from Sigma Aldrich and diluted to obtain 10% stock solutions by weighing (*w*/*v*). A sodium Dodecyl Sulfate (SDS) 10% solution was purchased from Invitrogen (Waltham, MA, USA, 15553027). All subsequent dilutions annotated in figures were made from these stocks. Ammonium chloride solution (ACS) was from Stemcell Technologies (Vancouver, BC, Canada, 07800).

### 4.4. Cell Counting

Counting of red blood cells was performed by using a Countess II automated cell counter (Invitrogen) according to the suggested protocol from the manufacturer. Briefly, a 1% washed erythrocyte solution was diluted to ~10^5^ cells/mL and 0.4% Trypan blue (1:1 ratio) was added before 10 μL of the stained cell suspension was added to the Countess cell counting chamber slide. After a 30 s incubation, the slide was inserted into the instrument for automatic cell counting.

### 4.5. Flow Cytometry of Erythrocytes Treated with Live/Dead Stain

The Countess II automated cell counter (see above) was used to adjust the negative control sample to ~10^5^ cells/mL and to subsequently dilute all samples by the same factor. Then, 0.5 μL of live/dead amine-reactive dye 488 (Fisher Scientific, Waltham, MA, USA) was added to 0.5 mL cell suspension and incubated for 30 min protected from light. Subsequently, the samples were washed twice with PBS pH 7 containing 1% bovine serum albumin (BSA), and finally resuspended in PBS pH 7 with 1% BSA. Cells were next counted, and the fluorescence measured using the 533/30 nm filter in an Accuri C6 flow cytometer (BD Biosciences, Franklin Lakes, NJ, USA).

### 4.6. General Procedure of the Hemolysis Assays

As soon as possible after phlebotomy, the blood samples were centrifuged at 1700× *g* for 5 min. The supernatant was removed by aspiration and 2 mL of PBS pH 7 was added. The washing step was repeated three times or until the supernatant was clear. After final aspiration, the remaining pellet was diluted 1:100 in PBS pH 7 to obtain a 1% erythrocyte suspension (or 1:100, 1:50, and 1:20 for assays testing the effect of erythrocyte concentration). For experiments using whole blood, the washing steps were not performed, but downstream experimentation was identical. For experiments performed with 96-well plates, 50 μL of test compound, water, or detergent were mixed with 50 μL of blood sample (1% erythrocyte suspension or whole blood). For experiments using larger volumes, 250 μL of test compound, water, or detergent were mixed with 250 μL blood sample in an Eppendorf tube. Samples were subsequently incubated at 37 °C for 60 min (or 15, 30, 60, 90, or 120 min for assays evaluating the effect of incubation time). After incubation, the plates/tubes were centrifuged at 1700× *g* for 5 min, and then 50 μL of the supernatants were transferred to transparent, flat-bottom 96-well plates (Anicrin, A013418). Finally, absorption was measured at 405, 530, and 570 nm in a Victor Nivo Multimode microplate reader (Perkin Elmer, Waltham, MA, USA).

### 4.7. Data Management

All experimental results were managed using Graphpad Prism (Dotmatics, Boston, MA, USA) to generate figures/plots and to calculate statistics.

## Figures and Tables

**Table 1:** Calculated hemolysis ratios (in %) according to Equation (1) for AMPs 1, 2, and 3 (100 µM) when using 1% mouse, rat, rabbit, and human erythrocyte solution. Normalization was performed on data averaged from three experimental replicates each containing two technical replicates using 10% Triton X-100 and PBS samples as positive and negative controls, respectively.

Species	AMP 1	AMP 2	AMP 3
Mouse	29.2 ± 32	14.9 ± 14.9	50.1 ± 14.4
Rat	12.9 ± 1.7	5.2 ± 1.9	29.5 ± 8.2
Rabbit	7.1 ± 1.1	4.6 ± 0.7	53.2 ± 7.4
Human	22.3 ± 4	8.5 ± 1.4	37 ± 5.1

**Table 2:** Calculated hemolysis ratios (in %) according to Equation (1) for dH_2_O and AMPs 1, 2, and 3 when testing 1% erythrocytes from different human individuals as well as a sample consisting of pooled blood from all 10 individuals. Normalization was performed on data averaged from three experimental replicates, each containing two technical replicates, and using 10% Triton X and PBS samples as positive and negative control, respectively.

Human Ind.	dH_2_O	AMP 1	AMP 2	AMP 3
# 1	38 ± 12.3	21.9 ± 4	8.2 ± 1.3	36.4 ± 5
# 2	57.7 ± 12.2	23 ± 2.8	10 ± 0.7	39.3 ± 6
# 3	50.8 ± 8.8	23.7 ± 2.1	9.8 ± 0.8	49.4 ± 8.5
# 4	47.2 ± 5.6	21.3 ± 3	10.4 ± 0.9	43.6 ± 4.2
# 5	38.8 ± 12.1	19.7 ± 3.7	11 ± 1	38.3 ± 7.3
# 6	36.2 ± 10.8	19.6 ± 4.7	6.4 ± 0.7	45.7 ± 6.4
# 7	49.6 ± 15.2	18.8 ± 4	8.9 ± 0.8	37.7 ± 6.9
# 8	49.4 ± 16.9	21.7 ± 8.6	9.1 ± 1.1	45.6 ± 9.2
# 9	49.1 ± 22.5	22.9 ± 6.8	9.6 ± 1	42.1 ± 4.7
# 10	39.4 ± 10.7	25.1 ± 13	9.8 ± 0.7	50.3 ± 4.6
Pooled	32.6 ± 7.3	22.7 ± 6.6	12.4 ± 3.1	45.4 ± 4.8

**Table 3:** Calculated hemolysis ratios according to Equation (1) for AMPs 1, 2, and 3 (100 µM) on 1%, 2%, and 5% pooled human erythrocyte solutions. Normalization was performed on data averaged from three experimental replicates, each containing two technical replicates, using 10% Triton X-100 and PBS samples as positive and negative controls, respectively.

Calculated Hemolysis (%)	dH_2_O	AMP 1	AMP 2	AMP 3
1% erythrocytes	32.6 ± 7.3	22.7 ± 6.6	12.4 ± 3.1	45.4 ± 4.8
2% erythrocytes	27.1 ± 2.7	13.7 ± 4.3	5.6 ± 0.8	41.1 ± 3.2
5% erythrocytes	22.7 ± 9	10.8 ± 3.2	5.0 ± 3.3	45.7 ± 8.2

**Table 4:** Calculated hemolysis ratios according to Equation (1) for dH_2_O, AMPs 1, 2, and 3 (100 µM) incubated (15–120 min) with a 1% human erythrocyte solution. Normalization was performed on data averaged from three biological replicates, each containing two technical replicates, using 10% Triton X-100 and PBS samples as positive and negative control, respectively.

Incubation Time	dH_2_O	AMP 1	AMP 2	AMP 3
15 min	32.8 ± 12.1	18.5 ± 2.3	7.8 ± 1.1	54.8 ± 8.2
30 min	34.8 ± 12.7	20.9 ± 4	7.4 ± 1.2	56.6 ± 7.4
60 min	37.1 ±14.2	26.6 ± 4.7	7.8 ± 1.3	64.6 ± 6.8
90 min	40.1 ±13.9	26.9 ± 6	8 ± 0.8	63 ± 4.5
120 min	47 ± 13.6	30.6 ± 10.6	8.6 ± 1.4	74.3 ± 21.1

**Table 5:** Calculated hemolysis ratios according to Equation (1) for AMPs 1, 2, 3, melittin or polymyxin B (PMB) (all at 100 μM) as well as for dH_2_O or ACS from measurements at 405, 530 and 570 nm on human whole blood. Measurements from samples with 10% SDS (positive control) or 10% Triton X-100 (positive control at 570 nm) and PBS (negative control) were used as 100% and 0% hemolysis for the normalization. Normalization was performed on data averaged from three experimental replicates, each containing two technical replicates.

Wavelength	dH_2_O	ACS	AMP 1	AMP 2	AMP 3	Melittin	PMB
405 nm	98.2 ± 0.4	97.8 ± 1.3	3.3 ± 3.2	0.4 ± 0.2	15.5 ± 7.1	98.3 ± 1.4	3.1 ± 1.1
530 nm	38.4 ± 8.5	28.1 ± 4.5	0.4 ± 0.7	0.2 ± 0.4	2.7 ± 1.4	36.6 ± 8.7	3 ± 2.4
570 nm	62.8 ± 1.4	45.1 ± 7.7	0.4 ± 1.2	−0.1 ± 0.3	3.8 ± 2.1	54 ± 15.5	4.5 ± 1.8

**Table 6:** Calculated hemolysis ratios according to Equation (1) for AMPs 1, 2, and 3, melittin and polymyxin B (PMB) (all at 100 μM), as well as for dH_2_O and ACS from measurements at 405, 530, and 570 nm on 1% washed human erythrocytes. Measurements from samples with 10% Triton X-100 (positive control) and PBS (negative control) were used as 100% and 0% hemolysis for the normalization. Normalization was performed on data averaged from three experimental replicates, each containing two technical replicates.

Wavelength	dH_2_O	ACS	AMP 1	AMP 2	AMP 3	Melittin	PMB
405 nm	73.9 ± 7.5	94.7 ± 14.4	27.7 ± 4.6	8.2 ± 1.4	73.5 ± 14.4	84.4 ± 8.1	4.4 ± 0.3
530 nm	67.6 ± 8.7	88.5 ± 14.3	29.2 ± 8	5.2 ± 3	68.4 ± 15	78.4 ± 4.3	3.0 ± 4.7
570 nm	71.2 ± 10.3	89.7 ± 13.4	25.3 ± 5.9	4.8 ± 6.6	65.7 ± 11.6	79.8 ± 8.2	1.3 ± 4.7

**Table 7:** Calculated hemolysis ratios according to Equation (1) for dH_2_O, ACS, AMPs 1, 2, and 3 (100 μM) or polymyxin B (PMB–100 μM) from measurements at 405 nm on 1% washed human erythrocytes incubated in polystyrene or polypropylene tubes. Measurements from samples with 10% Triton X-100 (positive control) and PBS (negative control) were used as 100% and 0% hemolysis for the normalization. Normalization was performed on data averaged from three experimental replicates, each containing two technical replicates.

	dH_2_O	ACS	AMP 1	AMP 2	AMP 3	PMB
Polystyrene	43.1 ± 18.6	71.2 ± 13.4	23.8 ± 4.3	9.7 ± 3.4	66.7 ± 12.7	6.1 ± 3.1
Polypropylene	29.5 ± 6	52.3 ± 9.2	19 ± 0.7	8.2 ± 2.6	57.5 ± 13.2	3.2 ± 1

**Table 8:** Calculated hemolysis ratios according to Equation (1) for dH_2_O, ACS, and AMPs 1, 2, and 3 (100 μM) or polymyxin B (PMB–100 μM) from measurements at 405 nm on 1% washed human erythrocytes. Total volumes during incubation were 100 or 500 μL. Measurements from samples with 10% Triton X-100 (positive control) and PBS (negative control) were used as 100% and 0% hemolysis for the normalization. Normalization was performed on data averaged from three experimental replicates, each containing two technical replicates.

	dH_2_O	ACS	AMP 1	AMP 2	AMP 3	PMB
100 μL	43.1 ± 18.6	71.2 ± 13.4	23.8 ± 4.3	9.7 ± 3.4	66.7 ± 12.7	6.1 ± 3.1
500 μL	31.4 ± 10.7	56.7 ± 8.8	21.9 ± 1.2	8.8 ± 3.9	72.7 ± 10.4	6.1 ± 2.8

## Data Availability

Not applicable.

## Supplementary Materials

The following supporting information can be downloaded at: https://www.mdpi.com/article/10.3390/ijms24032914/s1.
