# Supplementary material for: Optimization of the Hemolysis Assay for the Assessment of Cytotoxicity
Source: Int J Mol Sci. 2023 Feb 2;24(3):2914. doi: 10.3390/ijms24032914 (PMC9917735; doi:10.3390/ijms24032914)
Supplement: Supplementary file 1 [file ijms-24-02914-s001.zip › ijms-2120438-supplementary.pdf]

SUPPLEMENTARY FIGS & TABLES

Table S1. p-Values of unpaired t-tests comparing hemoglobin measurements of erythrocytes from different species.

	1xPBS	10% Triton X-100	AMP 1	AMP 2	AMP 3
Mouse vs Rat	0.473637	0.662841	0.189905	0.073682	0.010814
Mouse vs Rabbit	0.007201	0.084571	0.028320	0.004215	0.670845
Mouse vs Human	0.016696	0.424936	0.315800	0.024534	0.021823
Rat vs Rabbit	<0.000001	0.361175	<0.000001	<0.000001	0.000804
Rat vs human	0.000011	0.712083	0.060207	0.004217	0.363745
Rabbit vs Human	0.048961	0.725461	0.000002	0.000009	0.001118

Table S2: p-Values of unpaired t-tests comparing hemoglobin measurements of erythrocytes from different species treated with different detergents, ACS (ammonium chloride solution) or dH_2O

Tween				
	0.01 %	0.1 %	1 %	10 %
**Mouse vs Rat**	0.015113	0.850785	0.081551	0.388188
**Mouse vs Rabbit**	0.137435	0.000354	0.000064	0.591195
**Mouse vs Human**	0.000001	<0.000001	<0.000001	0.019292
**Rat vs Rabbit**	0.076628	0.001870	0.000010	0.674598
**Rat vs human**	0.007294	0.000007	<0.000001	0.088566
**Rabbit vs Human**	0.000004	0.000021	0.000015	0.035067
**Triton X-100**				
	0.01 %	0.1 %	1 %	10 %
**Mouse vs Rat**	0.000688	0.044119	0.082696	0.662841
**Mouse vs Rabbit**	0.018688	0.000838	0.000833	0.084571
**Mouse vs Human**	0.000639	<0.000001	0.000004	0.424936
**Rat vs Rabbit**	0.096495	0.0556807	0.917973	0.361175
**Rat vs human**	0.148763	0.009678	0.057330	0.712083
**Rabbit vs Human**	0.024301	0.001143	0.000811	0.725461
**SDS**				
	0.01 %	0.1 %	1 %	10 %
**Mouse vs Rat**	0.051795	0.108960	0.002578	0.134255
**Mouse vs Rabbit**	0.931932	0.336782	0.000108	0.008006
**Mouse vs Human**	0.241790	0.160237	0.000023	0.13569
**Rat vs Rabbit**	0.127769	0.019862	0.876993	0.191394
**Rat vs human**	0.985357	0.004757	0.138530	0.082816
**Rabbit vs Human**	0.304309	0.829798	0.101532	0.304674

	ACS	dH_2O
**Mouse vs Rat**	**0.851998**	**0.004081**
**Mouse vs Rabbit**	**0.295420**	**0.950525**
**Mouse vs Human**	**0.288659**	**0.000047**
**Rat vs Rabbit**	**0.345265**	**0.006175**
**Rat vs human**	**0.734393**	**0.093211**
**Rabbit vs Human**	**0.039753**	**0.000199**

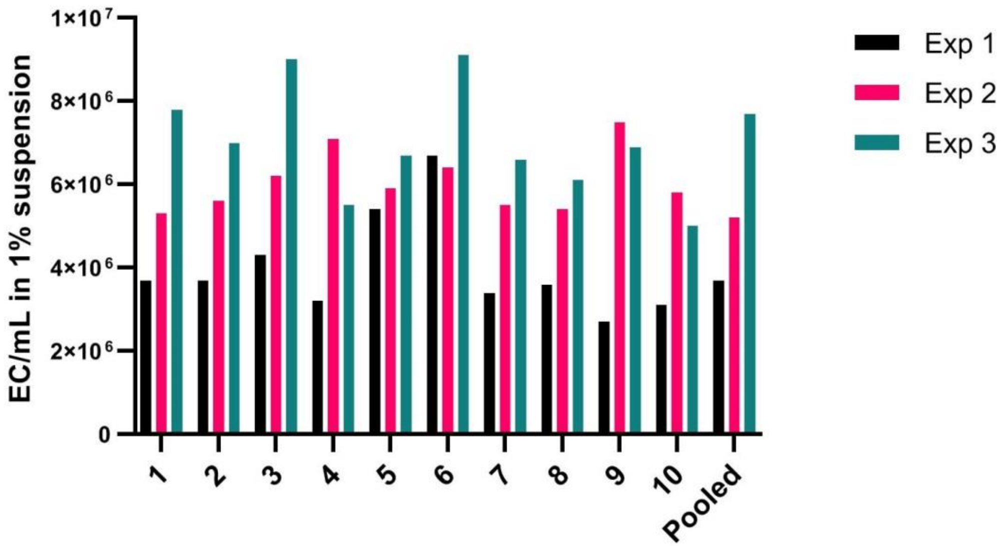

Figure S1: Erythrocyte count is similar for all human individuals

Erythrocyte (EC) count per mL (Y-axis) for ten human individuals, as well as sample pooled from all ten, when measured at day 1 (circles), 2 (squares) or 3 (triangles) after blood collection. The cells were counted by using a Countess II automated cell counter (see Methods section for details).

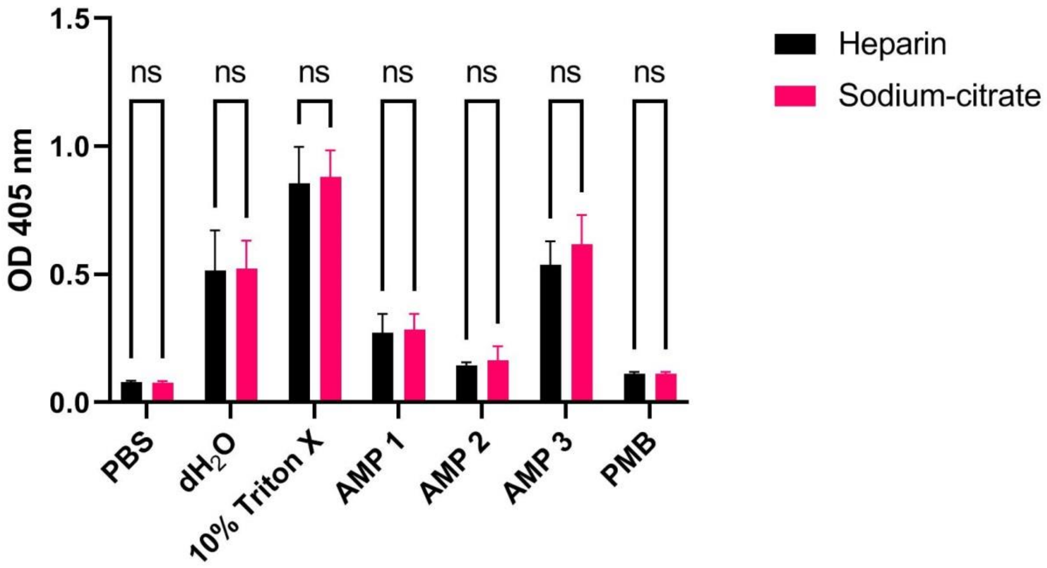

Figure S2: The anticoagulant does not affect hemolysis

OD measurements of free hemoglobin (Y-axis) from washed human erythrocytes (1%) collected in Heparin (black bars) or Sodium-citrate (red bars) anticoagulant tubes. The erythrocytes were incubated with PBS, dH_2O, Triton X-100 as well as with AMPs 1, 2, 3 or Polymyxin (PMB) (all at 100 μM) for 1 hour at 37°C. The measurements were performed at 405 nm. Average values from three experimental replicates, each containing two technical replicates, are presented with error bars (SD) included in plots. Significantly different data as defined from paired t-test are indicated by asterisks (p-values: *<0.05 **<0.01 ***<0.001 ****<0.0001 ns: non-significant).

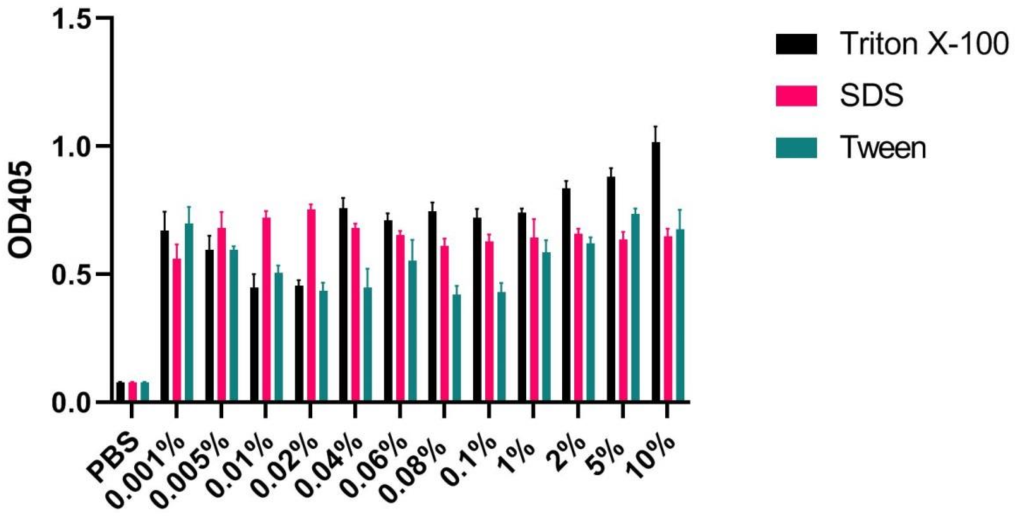

Figure S3: Hemoglobin measurements of human erythrocytes treated with Triton X-100, SDS

or Tween at concentrations ranging from 0.001% to 10%

OD measurements at 405 nm (Y-axis) of free hemoglobin in 1% human erythrocyte solution treated with different concentrations of Triton X-100 (black bars), SDS (red bars) and Tween (turquoise bars). Erythrocytes were incubated for 60 minutes at 37°C. Average values from three experimental replicates, each containing two technical replicates, are presented with error bars (SD) included in plots.

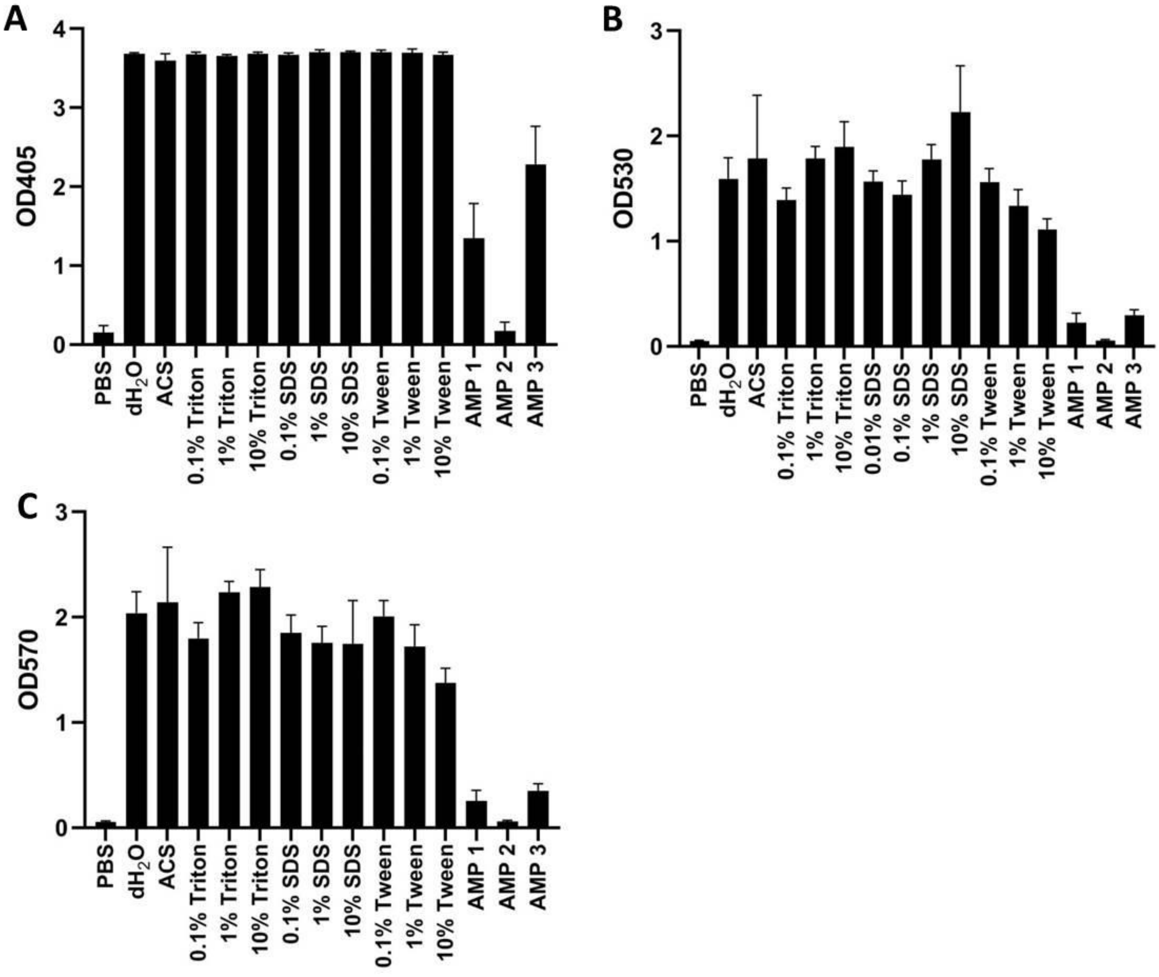

Figure S4: OD measurements at 405, 530 and 570 nm on rabbit whole blood

OD measurements of free hemoglobin in rabbit whole blood at 405 nm (A), 530 nm (B) or 570 nm (C). Samples were treated with PBS (negative control), AMPs (100 μM), dH_2O, ACS or different concentrations of Triton X-100, Tween or SDS. All samples were incubated for 1 hour at 37°C. Average values from three experimental replicates, each containing two technical replicates, are presented with error bars (SD) included in plots.

Table S3: Hemolysis ratios for rabbit whole blood when measured at 405, 530 or 570 nm

Wavelength	dH_2O	ACS	AMP 1	AMP 2	AMP 3
405 nm	99.8 +/- 0.5	97.5 +/- 2.4	34 +/- 12.3	0.6 +/- 3.2	60.4 +/- 14-5
530 nm	83.6 +/- 10.9	94 +/- 32.6	9.5 +/- 4.9	0.2 +/- 0.6	13.2 +/- 3
570 nm	88.8 +/-9.2	93.4 +/-23.5	8.9 +/-4.6	0.2 +/- 0.5	13.1 +/- 3.2

Calculated hemolysis ratios according to equation 1 for AMPs 1, 2 and 3 (100 μM) as well as for dH_2O and ACS from measurements at 405, 530 and 570 nm on rabbit whole blood. Measurements from samples with 10% SDS (positive control at 530 nm) or 10% Triton X-100 (positive control at 570 nm) and PBS (negative control) were used as 100% and 0% hemolysis, respectively, for the normalisation. Normalisation was performed on data averaged from three experimental replicates, each containing two technical replicates.

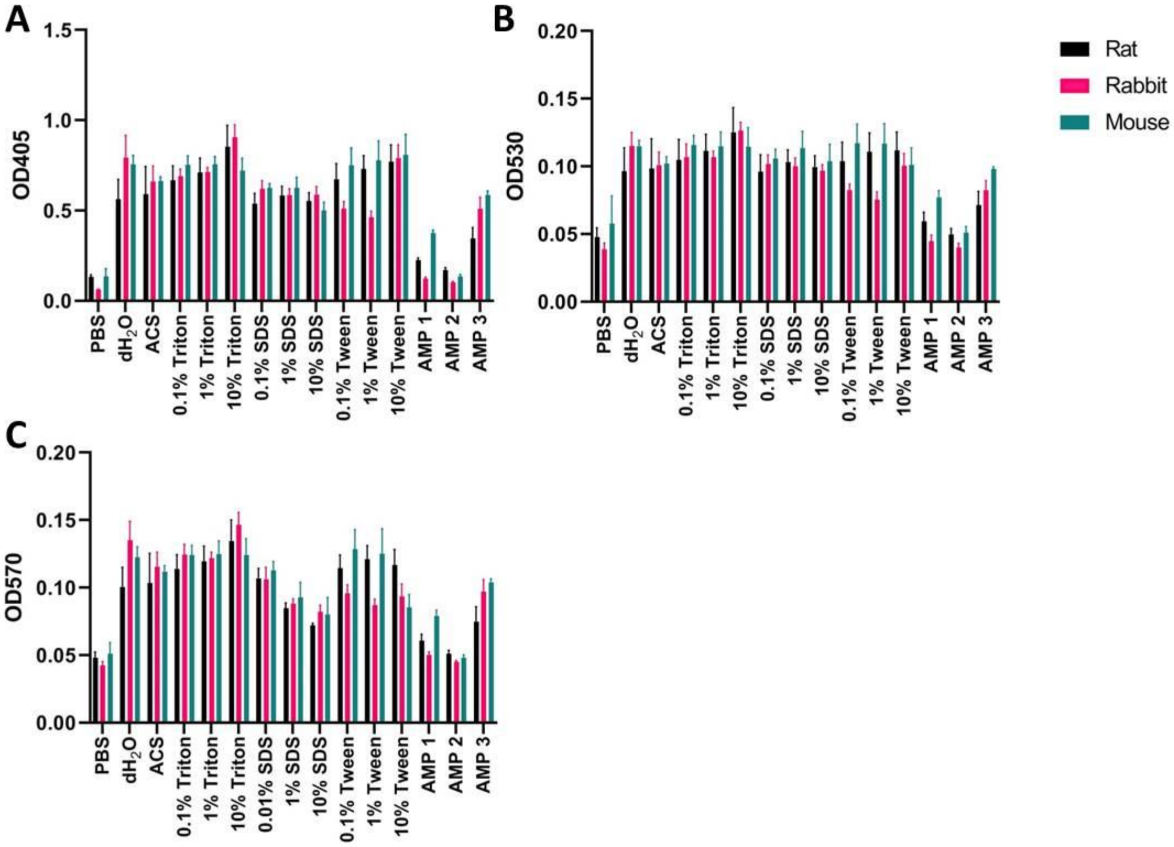

Figure S5: OD measurements at 405, 530 and 570 nm on rat, rabbit and mouse washed erythrocytes

OD measurements of free hemoglobin in washed erythrocytes from rat, rabbit or mouse at 405 nm (A), 530 nm (B) or 570 nm (C). Samples were treated with PBS (negative control), AMPs (100 µM), dH_2O, ACS or different concentrations of Triton X-100, Tween or SDS. All samples were incubated for 1 hour at 37°C. Average values from three experimental replicates, each containing two technical replicates, are presented with error bars (SD) included in plots.

Table S4: Hemolysis ratios of washed rat, rabbit and mouse erythrocytes when measured at 405, 530 or 570 nm

Rat

Wavelength	dH_2O	ACS	AMP 1	AMP 2	AMP 3
405 nm	59.6 +/- 15.2	63.4 +/- 21.3	12.9 +/- 1.7	5.2 +/- 1.9	29.5 +/- 8.2
530 nm	63.1 +/- 22.1	65.5 +/- 28.3	15 +/- 8.9	2.6 +/- 5.7	30.7 +/- 13.1
570 nm	60.8 +/- 16.7	64.1 +/- 25.3	14.7 +/- 5.5	3.3 +/- 3	31.1 +/- 12.7

Rabbit

Wavelength	dH_2O	ACS	AMP 1	AMP 2	AMP 3
405 nm	86.7 +/- 14.4	70.9 +/- 10.1	7.1 +/- 1.1	4.6 +/- 0.7	53.2 +/- 7.4
530 nm	87.1 +/- 11.3	70.8 +/- 11.1	6.8 +/- 5.2	1.7 +/- 3.6	50.1 +/- 7.7
570 nm	88.9 +/- 13.5	70.4 +/- 10.1	7.2 +/- 2.3	2.2 +/- 1.4	52.7 +/- 8.2

Mouse

Wavelength	dH_2O	ACS	AMP 1	AMP 2	AMP 3
405 nm	107 +/- 8.3	90.2 +/- 3.8	40.9 +/- 3	0.1 +/- 2	77.1 +/- 3.8
530 nm	101.2 +/- 7.5	78.6 +/- 8.7	34.5 +/- 8.7	-11.8 +/- 8	71.1 +/- 3.2
570 nm	64.6 +/- 7	55.1 +/- 3.8	25.5 +/- 3.7	-2.7 +/- 1.9	47.7 +/- 2.5

Calculated hemolysis ratios according to equation 1 for AMPs 1, 2, and 3 (100 μM) as well as for dH_2O and ACS from measurements at 405, 530 and 570 nm on washed erythrocytes from rat, rabbit and mouse. Measurements from samples with 10% Triton X-100 (positive control) and PBS (negative control) were used as 100% and 0% hemolysis for the normalisation. Normalisation was performed on data averaged from three experimental replicates, each containing two technical replicates.

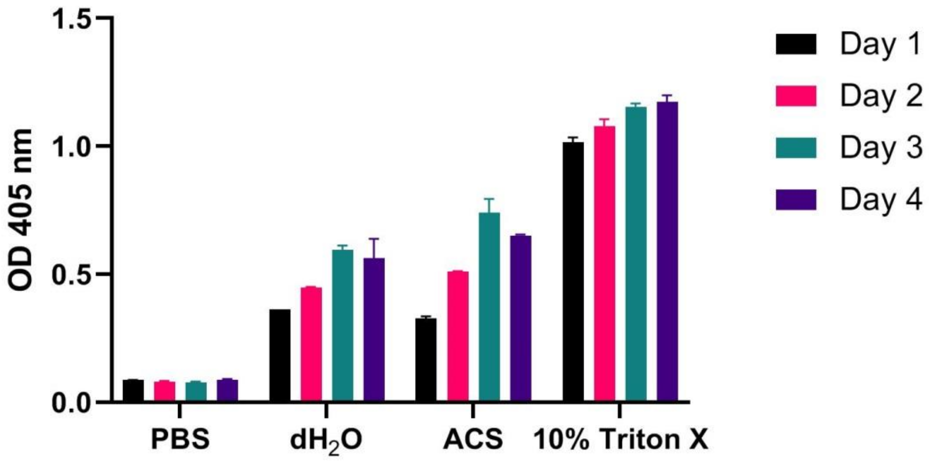

Figure S6: Effect of storage time of washed erythrocytes on fragility and hemolysis

OD measurements at 405 nm (Y-axis) of free hemoglobin in 1% human erythrocyte solution used for experimentation 1, 2, 3 or 4 days after blood collection. The washed erythrocytes were treated with PBS, dH_2O, ACS or 10% Triton X-100 for 60 minutes at 37°C. Average values from two technical replicates per day are presented with error bars (SD) included in plots.

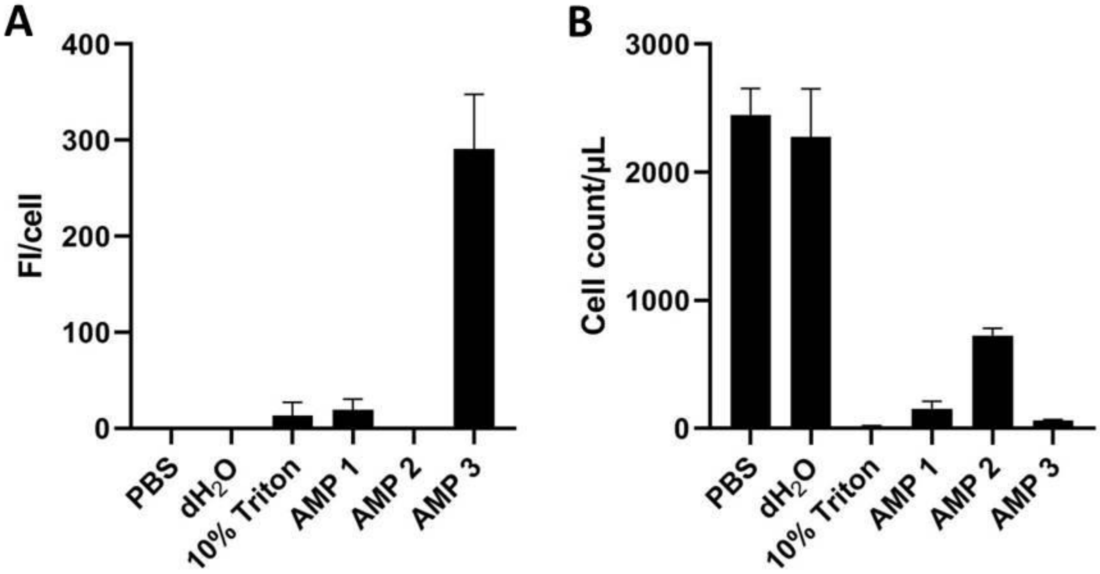

Figure S7: Samples treated with amine reactive LIVE/DEAD stain in order to distinguish whether intact cells are alive

Fluorescence intensity (FI) per cell (A) and cell count per µL (B) for washed human erythrocytes treated with PBS, dH_2O, 10% Triton X-100 or AMPs 1, 2 or 3 (100 µM) for 60 minutes and stained with LIVE/DEAD amine reactive dye (Fisher Scientific) measured at 530 nm with flow cytometry. The cell concentration was adjusted to 10^5/mL and cells were stained according to the protocol from Invitrogen (1 µL dye per 1 mL cell solution and washed with PBS containing 1% BSA) prior to flow cytometry (see Methods section).

Table S5: Properties of antimicrobial compounds

Name	Sequence	Hydrophobicity (peptide2.com)	Minimum inhibitory concentration (*E. coli*)
Mellitin	Gly-Ile-Gly-Ala-Val-Leu-Lys-Val-Leu-Thr-Thr-Gly-Leu-Pro-Ala-Leu-Ile-Ser-Trp-Ile-Lys-Arg-Lys-Arg-Gln-Gln	57.7 %	8 μM
AMP 1	Met-Asn-Leu-Val-Asp-Arg-Ala-Ile-Leu-Ile-Arg-Lys-Arg-Arg-Arg-Ala-Ala-Leu-Gln-Leu-Arg-Asp-Lys-Val-Leu-Arg-Tyr-Leu-Lys	48.3%	2 μM
AMP 2	Met-Lys-Arg-Lys-Lys-Lys-Ile-Leu-Ile-Lys-Lys-Val-Leu-Lys-Leu-Lys-Ser-Ser-Ala-Tyr	40.0%	4 μM
AMP 3	Met-Thr-Asp-Gly-Arg-Tyr-Leu-Ile-Lys-Arg-Val-Lys-Lys-Lys-Lys-Lys-Ala-Val-Leu-Gln-Leu-Ile-Leu-Lys-Phe-Leu	46.2%	4 μM
